# Effects of high-flow oxygen therapy on oxygenation in dogs undergoing diagnostic bronchoscopy

**DOI:** 10.3389/fvets.2025.1545427

**Published:** 2025-03-24

**Authors:** Julia Ortlieb, Hannah Bender, Matthias Schneider, Sabine Tacke, Esther Hassdenteufel

**Affiliations:** Department of Veterinary Clinical Sciences, Small Animal Clinic, Justus-Liebig-University Giessen, Giessen, Germany

**Keywords:** high flow oxygen therapy, oxygen supplementation, oxygenation, bronchoscopy, hypoxemia, dogs

## Abstract

**Introduction:**

Hypoxemia is a common complication during bronchoscopy and bronchoalveolar lavage (BAL). High-Flow Oxygen Therapy (HFOT) has been used to improve oxygenation and prevent periods of hypoxemia in people undergoing bronchoscopy.

**Objective:**

The main objective of this study was to evaluate the effect of HFOT on oxygenation in dogs undergoing diagnostic bronchoscopy compared to a traditional oxygen supplementation method (TOT). A secondary objective was to assess potential HFOT-related complications.

**Methods:**

Prospective randomized clinical trial. Dogs presented for diagnostic bronchoscopy were randomly assigned to receive either HFOT or TOT using nasal cannulas during the bronchoscopic procedure. Oxygenation was monitored through PaO_2_ measurements taken at seven time points: baseline (t0), after preoxygenation (t1), post-induction (t2), pre- and post-BAL sampling (t3 and t4), at the end of the procedure (t5), and 1 h after bronchoscopy (t6). Pre- and post-procedure thoracic radiographs were assessed for air leak syndrome or aerophagia.

**Results:**

20 privately owned dogs presented for diagnostic bronchoscopy were included in the study (HFOT group: *n* = 10, TOT group: *n* = 10). Baseline characteristics and physiological parameters did not differ significantly between groups. Five dogs in each group showed hypoxemia (PaO_2_ < 80 mmHg) at baseline with 1/5 in each group having PaO_2_ < 60 mmHg. HFOT improved oxygenation throughout the procedure, with a significant increase in PaO_2_ observed after preoxygenation (*P* = 0.001) and at the end of the procedure (*P* = 0.013). Additionally, only 1/10 dogs in the HFOT group experienced hypoxemia during bronchoscopy compared to 5/10 dogs in the TOT group, and patients in the HFOT achieved numerically higher PaO_2_ values across all time points during the procedure (t1–t5). No serious adverse events related to HFOT were observed, although aerophagia occurred in both groups without necessitating intervention.

**Conclusion:**

HFOT can improve oxygenation and prevent episodes of hypoxemia in dogs undergoing bronchoscopy compared to traditional oxygen supplementation methods.

## Introduction

Bronchoscopy serves as an essential diagnostic and therapeutic tool for patients suffering from respiratory diseases. Although the overall complication rate is low, patients undergoing bronchoscopy are exposed to an increased risk of developing hypoxemia, especially during bronchoalveolar lavage [BAL; (1–3)]. While specific incidence rates of hypoxemia during bronchoscopy in dogs are lacking, studies in human medicine report an incidence ranging from 30 to 70% (4), highlighting the need for optimized oxygenation strategies, particularly in patients with pre-existing respiratory compromise.

Different methods for oxygen supplementation exist in veterinary medicine; however, there is still considerable room for improvement in the options available during bronchoscopy, particularly in small patients. The placement of an endotracheal tube provides the safest method for ensuring adequate oxygenation by allowing controlled mechanical ventilation (5, 6). However, a sufficiently large enough tube is required, and this method is therefore not applicable to small dogs or cats. As an alternative, oxygen can be administered directly through the biopsy channel of the bronchoscope (7, 8), providing that it is not needed otherwise. Short episodes of hypoxemia are thus common during bronchoscopy in small animals and have to be anticipated. Other traditional oxygen supplementation methods, such as nasal or nasopharyngeal catheters or nasal cannulas, have been used anecdotally during bronchoscopy, however, no studies have yet evaluated their use of efficacy in veterinary patients.

High-Flow Oxygen Therapy (HFOT) emerged in the 2000s as an alternative oxygen supplementation method in pediatric patients (9, 10). Compared to other oxygen supplementation methods, the main difference and advantage are that HFOT delivers heated and humidified medical gas to the patient via nasal cannulas. This preconditioning of the gas allows for much higher flow rates without causing discomfort, as no drying or causative damage to the respiratory mucosa occurs compared with traditional oxygen therapy methods. Furthermore, HFOT devices contain an air-oxygen blender, allowing the delivered oxygen concentration to be adjusted between 21 and 100%, independent of the flow rate (9). In dogs, pharyngeal FiO_2_ levels of 95% have been measured at flow rates above 1 L/kg/min, whereas traditional nasal cannula oxygen therapy provides variable FiO_2_ levels, ranging from ~30 to 70%, depending on flow rate and patient factors (11, 12). This highlights the ability of HFOT to deliver a substantially higher and more consistent FiO_2_ compared to traditional methods.

Besides its ease of use and good patient tolerance, HFOT has been shown to have several beneficial effects, such as improving oxygenation, decreasing dead space through carbon dioxide washout, improving mucociliary clearance, decreasing work of breathing and even providing low levels of positive airway pressure (13). The increasing utilization of HFOT has also led to several studies evaluating its effectiveness in preventing hypoxemia during bronchoscopy in people with positive results (14–16).

HFOT in veterinary medicine is still in its infancy, with only a few studies evaluating its use, tolerance, and safety in dogs (11, 17–24). All studies to date have demonstrated that HFOT is well-tolerated, safe, and capable of improving oxygenation in dogs suffering from hypoxemia due to various conditions.

Despite its benefits, complications such as pneumothorax or pneumomediastinum could theoretically occur due to the high flow rates and variable airway pressures achieved with HFOT. However, complications in veterinary patients appear to be equally rare as in humans, with air leak syndrome directly caused by HFOT having been reported in only one veterinary study (25). The most common adverse event reported in three veterinary studies was the occurrence of aerophagia (11, 18, 19). Unfortunately, the current literature regarding HFOT-related complications in both human and veterinary medicine is minimal.

Based on the existing evidence in both people and dogs, HFOT represents an interesting alternative for preventing hypoxemia in veterinary patients undergoing bronchoscopy. Currently, only one small case series in dogs (23) and one pilot study in cats and dogs (24) have evaluated the use of HFOT during bronchoscopy using pulse oximetry to detect hypoxemia. To the author's knowledge, no prospective veterinary study has utilized arterial blood gas analysis as the gold standard for evaluating oxygenation during bronchoscopy in dogs.

The present study aims to address this knowledge gap, hypothesizing that HFOT will be a safer and more effective method to improve oxygenation and prevent hypoxemic events in dogs undergoing bronchoscopy, ultimately enhancing patient safety. The primary objective of this study was to evaluate the effects of HFOT on oxygenation during fiberoptic bronchoscopy and BAL sampling in dogs, compared to oxygen supplementation with a traditional method. The secondary objective was to assess the safety of HFOT by monitoring adverse events occurring with both methods.

## Materials and methods

### Study design

The study was conducted as a prospective randomized clinical trial, comparing High-Flow oxygen therapy with a traditional oxygenation method during flexible bronchoscopy in dogs. The study protocol was ethically approved by the responsible official regional authority (Regierungspraesidium Giessen) and registered under the trial number (No V 54 – 19 c 20 15 h 02 GI 18/17 kTV 12/2020).

### Patients

All client-owned dogs presented to the Small Animal Clinic of the Justus-Liebig-University of Giessen requiring fiberoptic bronchoscopy with bronchoalveolar lavage (BAL) were eligible for study inclusion.

The decision to perform fiberoptic bronchoscopy and sample collection was left to the discretion of the primary treating internal medicine clinician and was not part of the study itself.

Inclusion criteria for the study were a body weight of at least three kilograms, the presence of an indwelling arterial catheter, a baseline arterial blood gas analysis on ambient air, and initial thoracic radiographs. The initial arterial blood gas analysis and thoracic radiographs were both part of the routine workup and performed before bronchoscopy.

Failure to breathe spontaneously and requiring intubation with escalation to manual or mechanical ventilation was an exclusion criterion. Additionally, patients were excluded if an arterial catheter could not be placed or if the catheter dislocated and not all arterial blood gas analyses could be obtained.

After study inclusion, patients were allocated to receive either HFOT or TOT during bronchoscopy. A computer-generated block randomization was used to assign patients and ensure balanced allocation across groups.

Soft silicone nasal cannulas with double prongs were used as a patient interface for oxygen supplementation in both groups. The prongs were inserted into the nostrils, and the tubing was secured behind the patient's head. The cannula size was selected, ensuring that not more than 50% of the nares would be occluded.

Patients in the HFOT group received oxygen using the Vapotherm Precision Flow^®^ device (Vapotherm Inc., Exeter, USA) with the matching nasal cannulas (High-Velocity Nasal Insufflation (HI-VN^®^) Cannula (for use with Precision Flow), Intermediate Infant/Infant/Pediatric-Small/Pediatric-Adult-Small, Vapotherm Inc., Exeter, USA) as patient interface. Selected High-Flow settings consisted of a flow of 1 L/kg/min, a temperature of 35°C, and a FiO_2_ of 100%. Flow settings were based on a previous study evaluating the effect of HFOT in dogs (11). The High-Flow device was turned on at least 10 min before connecting the patient to ensure the air was sufficiently warmed and humidified.

Patients enrolled in the control group received oxygen supplementation following current procedures used in our clinic. Pure oxygen was humidified using a standard bubble humidifier (Kendall™, Sterile Water for Inhalation, Covidien, Mansfield, USA) and delivered to the patients via an oxygen line attached to a nasal cannula. If the standard nasal cannula (Nasal oxygen cannula with tubing, Centramed, Koblenz, Germany) did not fit the patient's nares size, the HI-VN^®^ cannulas from the HFOT group were used. Oxygen flow for the TOT group was set at 200 ml/kg/min using a standard flow meter (MediFlow^®^Ultra II, GCE Mediline, Chotebor, Czech Republic). As there are no studies on the use of nasal prongs in dogs, the flow rate from a study by Dunphy et al. (12) evaluating nasopharyngeal catheters was used, as this was assumed to most closely resemble the conditions with nasal prongs. According to this study, flow rates of 100 ml/kg/min per catheter are recommended and can provide an FiO_2_ of ~60% when bilateral catheters are used.

### Bronchoscopic procedure

All patients received 0.2 mg/kg butorphanol intravenously ~15 min before induction of anesthesia. The dogs were then placed on the bronchoscopy table and allowed to rest in sternal recumbency until the medication took effect.

**Figure 1:**
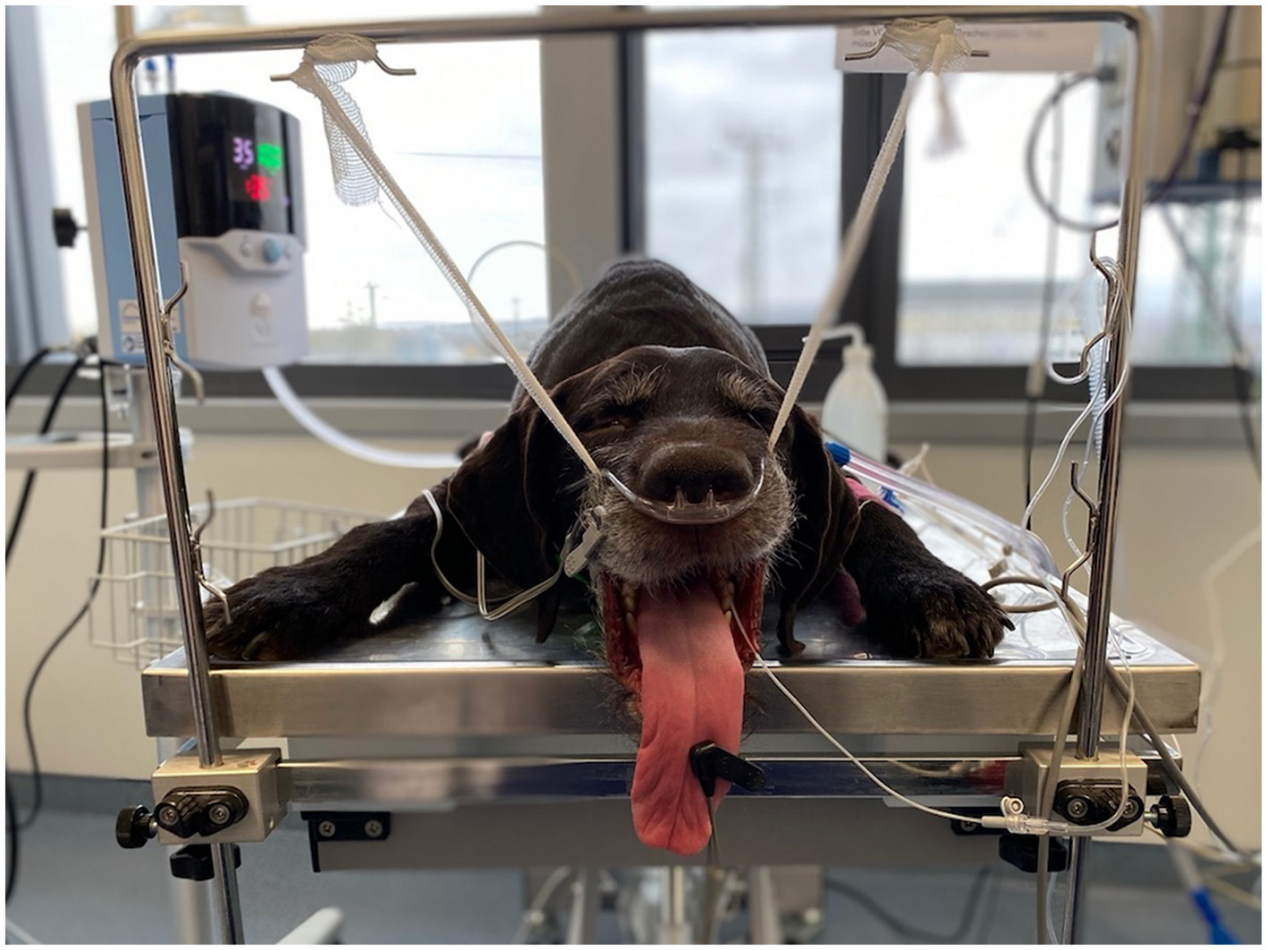
Picture depicting patient placement and setup for bronchoscopy.

Patients in both groups received preoxygenation for 5 min using the allocated oxygen delivery method before induction of anesthesia. For general anesthesia, 0.5 mg/kg diazepam and 2–4 mg/kg propofol titrated to effect were used in all patients. After induction, the patients were placed in sternal recumbency, and the patients' upper jaws were suspended from a specific suspension device using a gauze bandage to ensure the mouth was kept ajar throughout the bronchoscopy (Figure 1). The allocated oxygen delivery method remained attached to the patients the entire time to ensure a continuous oxygen supply.

After the patients were positioned correctly, a flexible fiberoptic bronchoscope was inserted, and the airways were endoscopically examined by the primary treating internal medicine clinician. After visual examination, the bronchoscope was advanced and wedged into a lower bronchial segment, where bronchoalveolar lavage was performed by administering ~0.5 ml/kg sterile saline solution, followed by immediate aspiration. During the bronchoscopic procedure, anesthesia was maintained via propofol constant rate infusion with 0.1 mg/kg/min. With the completion of the fiberoptic bronchoscopy, the constant rate infusion was discontinued, while oxygen supplementation was continued in all patients until they were awake and able to lift their heads.

As soon as the patients could lift their heads, oxygen supplementation was stopped, and the nasal cannulas were removed. Subsequently, repeat thoracic radiographic images were taken to assess the extent of any adverse effects, and the patients were then transferred to the appropriate ward or ICU for further monitoring and care.

Final diagnoses were made by the primary internal medicine clinician based on the available findings of physical examination, laboratory diagnostics, cytology, and bacteriological culture and were not part of the study design.

### Data collection

Baseline characteristics obtained for all patients included breed, age, weight, sex, indication for bronchoscopy, and vital signs (heart rate, respiratory rate, temperature). An indwelling arterial catheter was placed in either the left or right dorsal pedal artery under sterile conditions during the initial workup, and a baseline arterial blood gas analysis on ambient air was performed for each patient.

During the entire anesthesia, vital signs including heart rate, respiratory rate, and body temperature, as well as ECG and SpO_2_ (pulse oximetry), were continuously monitored and recorded every minute using a Carescape B650 monitor (Carescape B650 Monitor, General Electric Healthcare, Chicago, USA).

#### Arterial blood gas analyses

**Figure 2:**
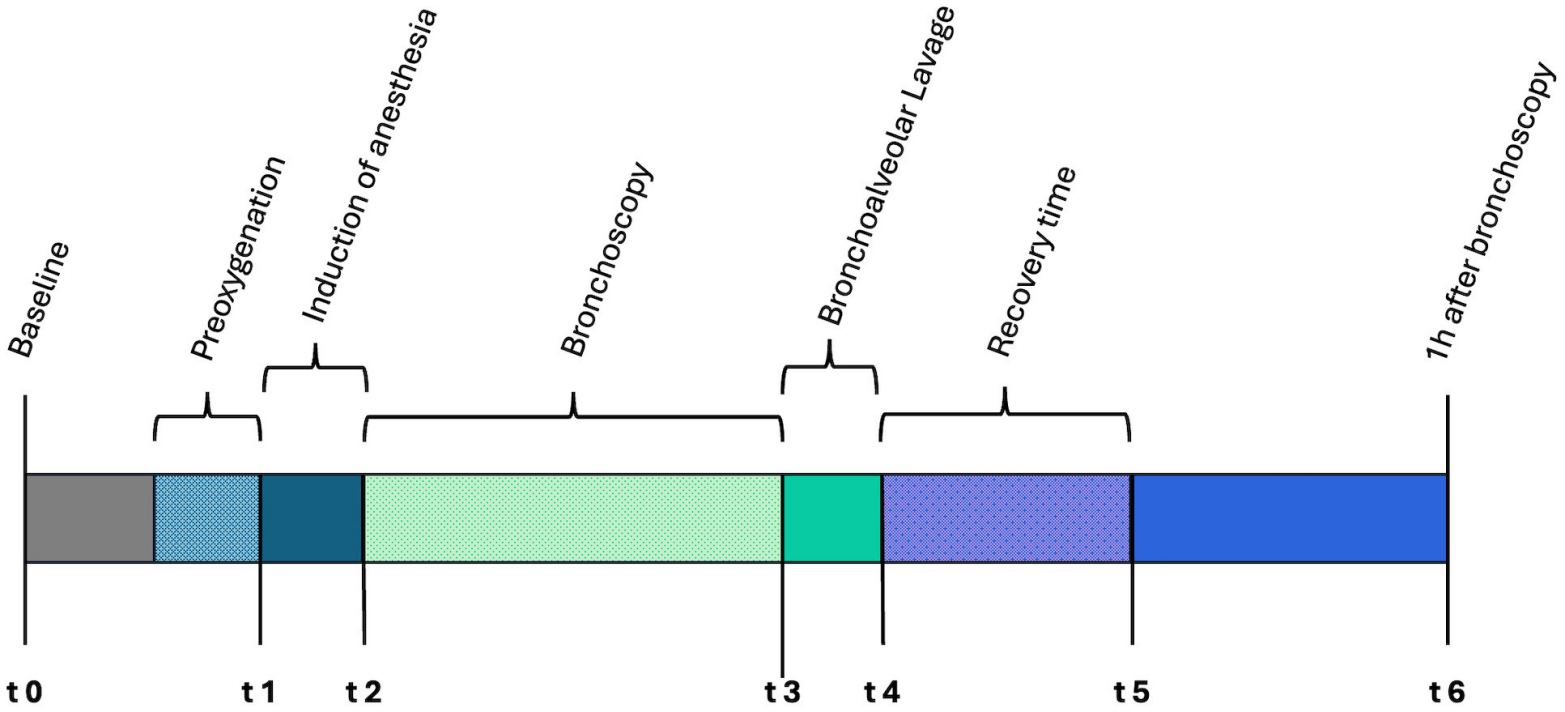
Timeline depicting the bronchoscopic procedure and the individual time points for arterial blood gas sampling.

Several arterial blood gas analyses were performed at predetermined time points (t0–t6) to assess oxygenation at critical stages throughout the procedure (Figure 2).

For blood sampling, 1 ml heparinized arterial collection syringes (BD A-Line, Arterial Blood Collection Syringe, Becton, Dickinson and Company, Plymouth, United Kingdom) were used, and a minimum of 0.3 ml of blood was drawn, depending on the patient's size. Samples were analyzed immediately after blood collection using the COBAS blood gas analyzer (Cobas^®^ b 123 System, Roche Diagnostics Deutschland GmbH, Mannheim, Germany). Heart rate, respiratory rate, and rectal temperature were noted at the time of each blood collection.

The first arterial measurement (t0) was performed on ambient air as part of the diagnostic workup. Subsequent measurements were taken 5 min after the start of preoxygenation (t1), immediately after anesthesia induction (t2), immediately before (t3) and directly after BAL (t4), and immediately before the discontinuation of oxygen supplementation (t5). The final arterial blood gas measurement was taken 1 h after the last measurement while breathing room air (t6; Figure 2).

#### Complications and adverse effects of oxygen therapy

Thoracic radiographs were performed before and immediately after bronchoscopy to evaluate the occurrence of adverse effects such as gastric distension or pneumothorax. Initial radiographs were then compared with the subsequent radiographs to detect changes resulting from oxygen supplementation. Any abnormality, such as esophageal and gastric distension, pneumothorax, or pneumomediastinum, was recorded for each patient. Additionally, if patients showed signs of aerophagia, the localization of the distension (esophageal, gastric, or both) and severity were also recorded. Since no official grading system for aerophagia exists, severity was graded as a) mild if only slight signs of aerophagia were visible, b) moderate if distension of the stomach reached the caudal rib, and/or if air was present in one-third of the esophagus, and c) severe, if gastric distension was visible beyond the contour of the caudal rib and/or if air was present in more than one-third of the esophagus.

### Statistical analysis

Statistical analysis was performed using commercially available statistical software (GraphPad Prism 9, GraphPad Software LLC., San Diego, California, USA). Data distribution was assessed for normality using the Shapiro-Wilk test. Normally distributed data were expressed as mean and standard deviation, while non-normally distributed data were expressed as median and range. Unpaired student's *t*-tests were used to compare data between the groups at each individual time point and were depicted as box-and-whisker plots with Tukey's method. Additionally, a two-way repeated measures ANOVA was applied to assess the main effects of time and oxygen supplementation method, as well as their interaction. *Post-hoc* multiple comparisons using Tukey's correction were performed to compare individual time points between groups when the main effects were significant. A linear graph using the mean and standard error of mean was plotted to illustrate changes in PaO_2_ over time and between groups. Statistical significance was set at *p* < 0.05.

## Results

### Patients

Between May 2020 and June 2022, 23 dogs met the inclusion criteria for participation in the study. Three dogs had to be excluded because arterial blood sampling could not be obtained at all specified time points due to a dislocation of the indwelling arterial catheter in all cases. Eventually, a total of 20 dogs were included in the study, with 10 dogs represented in each of the groups. There was no significant difference in weight, age, or gender between both groups, and no specific breed was distinctly present in the study population.

The TOT group consisted of two mixed-breed dogs and eight purebred dogs. Breeds included one of each of the following: Jack Russel Terrier, Akita Inu, Magyar Vizsla, Wirehaired Dachshund, German Longhaired Pointer, Chihuahua, Pyrenean Mountain dog, and Golden Retriever. Median age was 10 years (range from 1 to 12 years), and the median weight was 19.75 kilograms (range from 4.4 to 47.8 kg).

The HFOT group included three mixed-breed dogs and seven purebred dogs. Breeds presented were one each of the following: Husky, Rhodesian Ridgeback, Cocker Spaniel, Airedale Terrier, Wirehaired Dachshund, White Shepherd, and German Wirehaired Pointer. The median age of patients within the HFOT group was 7.5 years (range from 2 to 13 years), and the median weight was 22.85 kilograms (range from 6.4 to 45.0 kg). Patient characteristics for both groups are listed in Table 1.

**Table 1:** Patient characteristics and physiological parameters at baseline.

**Parameter**	**TOT**	**HFOT**	**P-Value**
Number of patients	10	10	
**Gender**			
Female	3 (30%)	3 (30%)	
Female neutered	2 (20%)	2 (20%)	
Male	2 (20%)	3 (30%)	
Male neutered	3 (30%)	2 (20%)	
Age (years)	10 (1–12)	7.5 (2–13)	0.270
Weight (kg)	19.75 (4.4–47.8)	22.85 (6.4–45)	0.878
Heart rate (beats/min)	99 ± 19	111 ± 17	0.164
Respiratory rate (breaths/min)	34 ± 17	33 ± 6	0.495
Body Temperature (°C)	38.4 ± 0.8	38.8 ± 0.4	0.130
PaO_2_ (mmHg)	76.5 ± 13	79.4 ± 15.6	0.664
P_a_CO_2_ (mmHg)	36.1 ± 3.7	34.3 ± 4.7	0.359
pH	7.397 ± 0.03	7.396 ± 0.03	0.976
PaO_2_/FiO_2_ (mmHg)	364.4 ± 61.8	377.9 ± 74.4	0.664
AaDO_2_	28 ± 12	28 ± 18	0.973

Data shown are listed as mean ± standard deviation, median, and range or as numbers and percentages. *P*-Values are shown for evaluated comparison between both groups. PaO_2_, partial pressure of arterial oxygen; P_a_CO_2_, partial pressure of arterial carbon dioxide; PaO_2_/FiO_2_, ratio of partial pressure of arterial oxygen to fraction of inspired oxygen; AaDO_2_, alveolar-arterial oxygen difference.

Flow rates of 1 L/kg/min were used according to the study design, however, one dog in the HFOT group had a body weight above 40 kg and thus received a lower flow rate than previously planned (~0.9 L/kg/min instead of 1 L/kg/min), as the Vapotherm Precision Flow has a maximum flow limit of 40 L/min.

### Baseline parameters

There were no significant differences between both groups regarding patient characteristics and baseline physiological parameters, including PaO_2_. *P*-Values for individual parameters are listed in Table 1. The main indication for bronchoscopy in all dogs was persistent chronic coughing, and the final diagnoses varied between all patients. Indications and final diagnoses are listed in Table 2.

**Table 2:** Main indications and final diagnoses.

**Parameter**	**TOT**	**HFOT**
Main indication for bronchoscopy:		
Chronic coughing	4	
Chronic coughing with exercise intolerance	1	6
Chronic coughing with exercise intolerance and chronic tachypnoea	1	1
Chronic coughing with intermittent fever	1	
Chronic progressive tachypnoea with acute coughing	2	2
Exercise-induced dyspnea and cyanosis with acute coughing	1	1
Final diagnosis:		
Laryngeal paralysis	2	
Tracheobronchomalacia	1	
Chronic bronchitis	1	2
Chronic bronchitis with bacterial pneumonia	1	
Chronic bronchitis with bronchial collapse	1	
Bronchial collapse with secondary pneumonia	1	
Pulmonary fibrosis with bronchial collapse		1
Bronchopneumonia	2	3
Eosinophilic bronchopneumopathy		1
Allergic pneumonitis		1
Multicentric high-grade lymphoma		1
Immune-related pneumonitis	1	
Fever-of-unknown-origin (FUO)		1

Numbers in the HFOT and TOT columns represent the number of dogs per group.

Five dogs in each group showed hypoxemia, defined as PaO_2_ < 80 mmHg, at baseline. Out of these dogs, one in each group showed severe hypoxemia with a PaO_2_ < 60 mmHg. The final diagnoses for the dogs with severe hypoxemia in the TOT and HFOT groups were tracheobronchomalacia and pulmonary fibrosis with bronchial collapse, respectively (Table 2). Both dogs were presented on the day of bronchoscopy and showed no distinct clinical signs indicating the severity of hypoxemia beforehand.

### Arterial partial pressure of oxygen (PaO_2_) at specified time points

#### PaO_2_ after preoxygenation (t1)

After preoxygenation, patients achieved significantly higher values of PaO_2_ in the HFOT group compared to patients receiving TOT (*p* = 0.001).

PaO_2_ within the TOT group showed an increase from 76.5 ± 12.9 mmHg at baseline (t0) to 200.3 ± 96.5 mmHg after preoxygenation (t1). Within the HFOT group, PaO_2_ increased from 79.4 ± 15.6 mmHg at baseline (t0) to 375.6 ± 95.3 mmHg after preoxygenation (t1).

**Figure 3:**
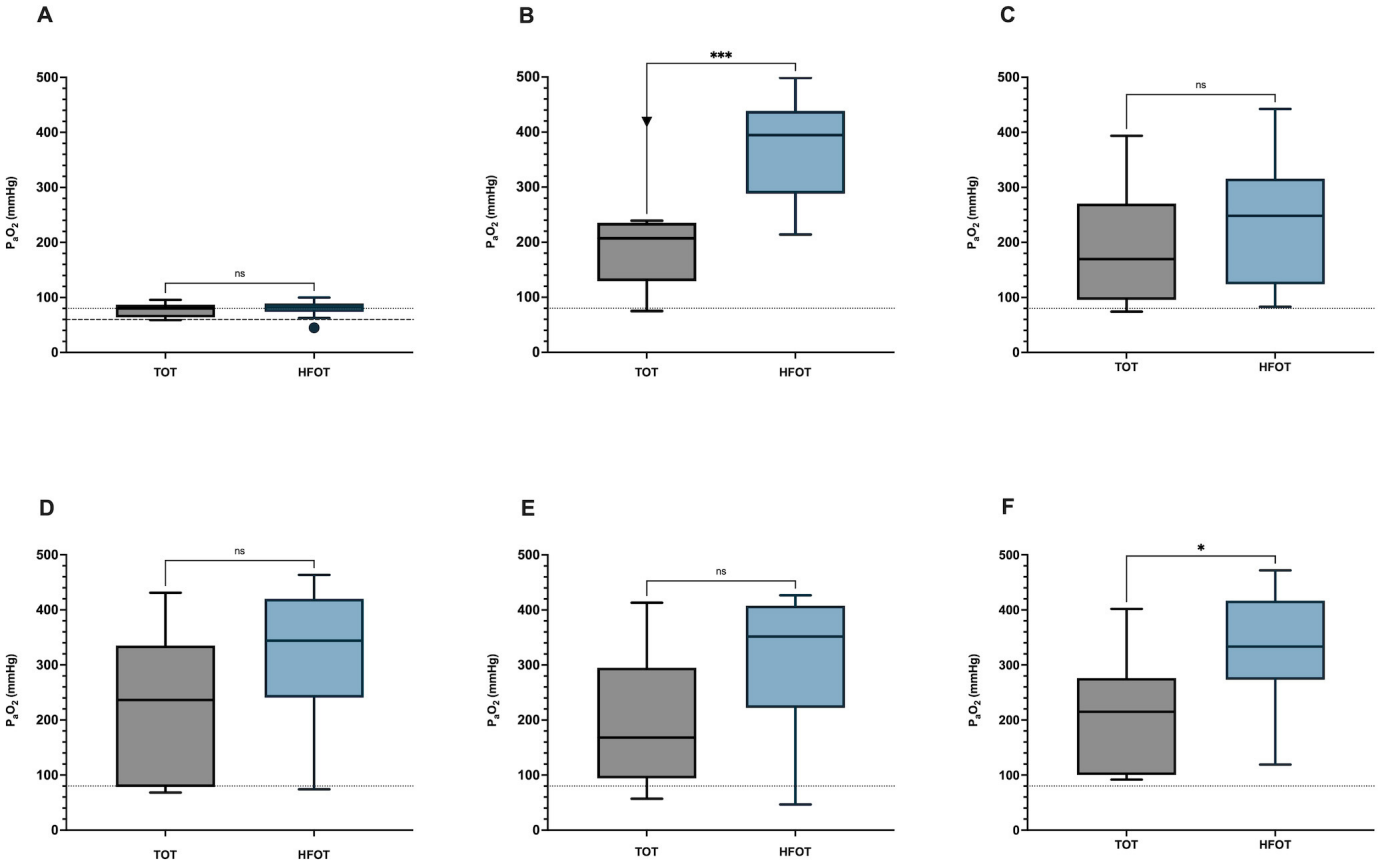
Differences in PaO_2_ at different time points between the TOT and the HFOT group. **(A)** Differences in PaO_2_ at baseline (t0); *p* = 0.664. **(B)** Differences in PaO_2_ after preoxygenation (t1); *p* = 0.001. **(C)** Differences in PaO_2_ after induction of anesthesia (t2); *p* = 0.304. **(D)** Differences in PaO_2_ before BAL (t3); *p* = 0.102. **(E)** Difference in PaO_2_ after bronchoalveolar lavage (t4). *p* = 0.056. **(F)** Difference in PaO_2_ at the end of the procedure (t5); *p* = 0.013. PaO_2_, partial pressure of arterial oxygen; TOT, traditional oxygen therapy; HFOT, High-Flow Oxygen Therapy. ^*^P ≤ 0.05; ^***^P ≤ 0.001; ns > 0.05.

Differences in PaO_2_ values between both groups after preoxygenation are shown in Figure 3.

#### PaO_2_ after induction of anesthesia (t2)

Nearly all patients showed a decrease in PaO_2_ after induction of anesthesia, except for two dogs in the TOT group, which showed an increase in PaO_2_. Both dogs were tachypneic or panting before induction, which might have affected measurement. Mean PaO_2_ after induction of anesthesia (t2) was 189.3 ± 103.4 mmHg in the TOT group and 241 ± 115 mmHg in the HFOT group. 1/10 dogs in the HFOT group showed a drastic drop from 413.1 mmHg after preoxygenation to only 82.9 mmHg after induction of anesthesia. The same dog was classified as severely hypoxemic at baseline and later diagnosed with pulmonary fibrosis. There was, however, no statistically significant difference in PaO_2_ after induction between both groups (*p* = 0.304). Differences in PaO_2_ values between both groups are shown in Figure 3.

#### PaO_2_ before BAL sampling (t3)

There was no statistically significant difference in PaO_2_ immediately before BAL between both groups (*p* = 0.102). Mean PaO_2_ in the TOT group was 218.3 ± 133.6 mmHg and 317.2 ± 128.8 mmHg in the HFOT group. Although no statistical difference could be established, the visual graphic analysis shows a trend toward higher PaO_2_ values within the HFOT group, as shown in Figure 3.

A decrease in PaO_2_ ≤ 80 mmHg was observed in 3/10 dogs in the TOT group and 1/10 dogs in the HFOT group at t3. The patient in the HFOT group was again the same dog with pulmonary fibrosis experiencing hypoxemia in previous measurements. All three dogs in the TOT group were also hypoxemic at baseline. All remaining dogs within both groups showed increased PaO_2_ from the previous measurement.

#### PaO_2_ after BAL sampling (t4)

In the TOT group, 5/10 dogs experienced a decrease in PaO_2_ after BAL, with one patient decreasing below 80 mmHg and one decreasing below 60 mmHg. The latter also showed a decrease in PaO_2_ before lavage sampling at t3 yet achieved values above 200 mmHg at time points t1, t2, and t5. The five remaining dogs in the TOT group showed a slight increase in PaO_2_ compared to values before BAL sampling. Mean PaO_2_ in this group after BAL was 192.4 ± 117.5 mmHg.

One dog within the HFOT group experienced severe hypoxemia immediately after BAL, decreasing from a previous PaO_2_ of 74.1 mmHg at t3 to 46.7 mmHg. Again, this was the dog with pulmonary fibrosis that had already shown inadequate oxygenation at baseline, after induction, and before BAL. Out of the remaining dogs in the HFOT group, six showed a slight decrease, and three showed a slight increase in PaO_2_ after bronchoalveolar lavage, yet none decreased below 140 mmHg, and mean PaO_2_ within the HFOT group was 305.0 ± 128.0 mmHg.

Statistical analysis barely failed to show a significant difference (*p* = 0.056), although a clear visual trend toward higher PaO_2_ values within the HFOT group can be seen in Figure 3.

#### PaO_2_ at the end of the procedure (t5)

There was a significant difference in PaO_2_ at the end of the procedure between the TOT group and the HFOT group (*p* = 0.013), as seen in Figure 3.

Mean PaO_2_ in the TOT group was 204.2 ± 103.4 mmHg and mean PaO_2_ in the HFOT group was 330.8 ± 102.3 mmHg. No dogs in either group experienced PaO_2_ ≤ 90 mmHg. The dog with pulmonary fibrosis showed adequate oxygenation with a PaO_2_ of 412.3 mmHg.

#### PaO_2_ 1 h post procedure (t6)

A comparison of PaO_2_ values obtained 1 h post-procedure showed no significant difference between the two groups (*p* = 0.975). Mean PaO_2_ in the TOT and HFOT groups were 86.7 ± 18.7 mmHg and 87.0 ± 17.0 mmHg, respectively. Although 6/10 dogs in the TOT group and 9/10 in the HFOT group showed an increase in PaO_2_ compared to baseline values, there was no significant difference between t0 and t6 for either group (TOT: *P* = 0.172, HFOT: *p* = 0.310).

#### Comparison of PaO_2_ at all specified time points

**Figure 4:**
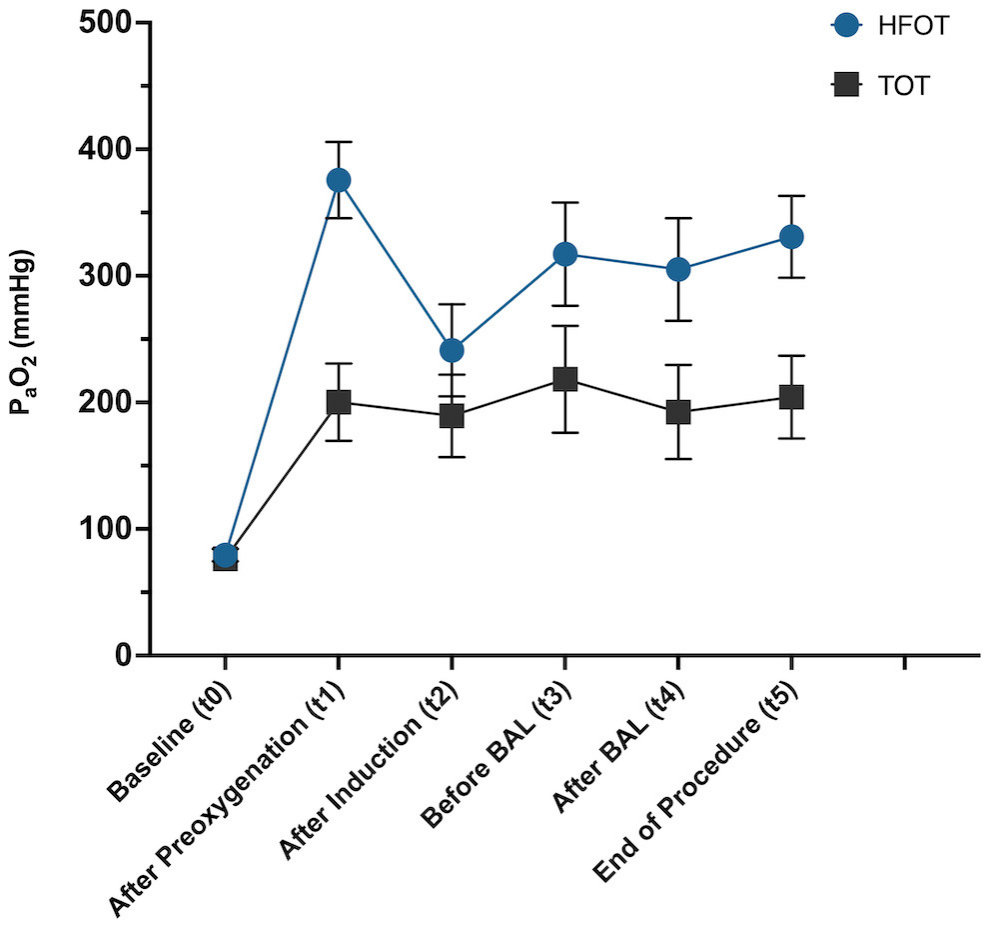
Linear graph showing mean and standard error of mean from baseline (t0) across all time points during the procedure (t1–t5) for each group, TOT, traditional oxygen therapy; HFOT, High-Flow Oxygen Therapy.

The two-way repeated measures ANOVA revealed significant interactions between time and oxygen supplementation method [TOT vs. HFOT; F_(5, 90)_ = 3.05, *p* = 0.01]. Both the main effect of time [F_(3, 55.4)_ = 20.1, *p* < 0.001] and the oxygen supplementation method [F_(1, 18)_ = 7.8, *p* = 0.01] were significant, demonstrating that the type of oxygen supplementation influenced oxygenation. Multiple comparisons revealed specific significant differences between the two methods at various time points. Notably, after preoxygenation (t1) and at the end of the procedure (t5), HFOT resulted in significantly higher oxygenation compared to TOT (mean difference = −175.3, 95% CI = −265.4 to −85.2, *p* = 0.001 and mean difference = −126.6, 95% CI = −223.2 to −30.00, *p* = 0.013, respectively). No significant differences were observed between the two methods at baseline (t0; mean difference = −2.84, *p* = 0.664, after induction (t2; mean difference = −51.8, *p* = 0.304), before BAL (t3; mean difference = −98.9, *p* = 0.109), and after BAL (t4; mean difference = −112.6, *p* = 0.055). However, while statistical significance was not achieved at these time points, numerical data and graphical presentation consistently show higher PaO_2_ values in the HFOT group compared to the TOT group at all time points as seen in Figure 4.

### Arterial partial pressure of carbon dioxide (PaCO_2_)

**Figure 5:**
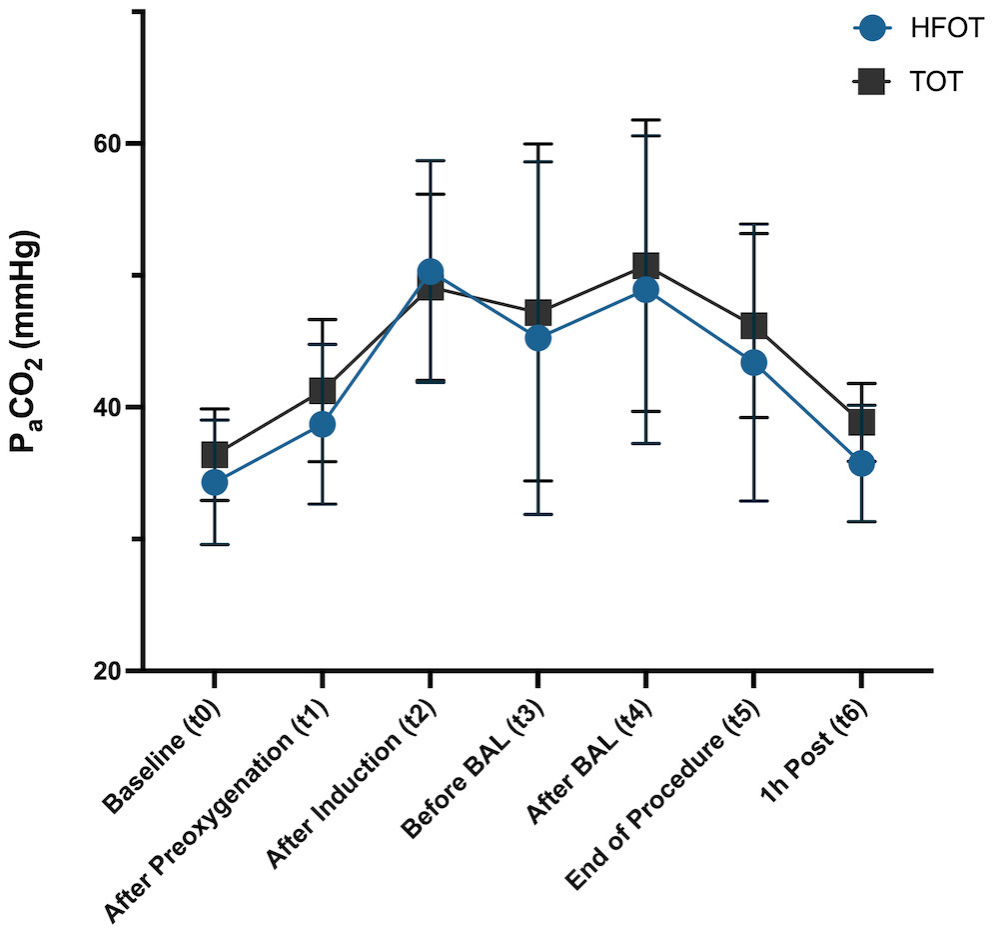
Difference in P_a_CO_2_ values (mean and standard deviation) between TOT and HFOT groups across time points t0–t6, TOT, traditional oxygen therapy; HFOT, High-Flow Oxygen Therapy.

There were no significant differences in PaCO_2_ between groups at all specified time points (t0–t6). 7/10 dogs in the TOT group and 6/10 in the HFOT group showed increased levels of PaCO_2_ between induction of anesthesia (t2) and the end of the procedure (t5). A maximum value of 64.4 mmHg was shown in one dog after BAL in the TOT group, while one dog in the HFOT group had maximum levels of 63.4 and 63.0 mmHg before and after BAL, respectively. None of the patients demonstrated elevated levels of PaCO_2_ 1 h post-procedure (t6). PaCO_2_ values did not achieve statistical significance at any time point when comparing effects of time and oxygen method or their interaction between the groups. Mean values across all time points are displayed in Figure 5.

### Complications and adverse events

Both oxygen delivery methods were well tolerated by all patients. Initial pawing at the nasal cannula during preoxygenation, potentially indicating discomfort, was observed in 1/10 dogs in the TOT group and 2/10 in the HFOT group. However, pawing subsided in all dogs after a few minutes of acclimatization to their designated oxygen delivery method.

None of the patients experienced severe complications during anesthesia, although several dogs in both groups showed hypoxemia on blood gas analysis. None of the patients showed signs of atelectasis, pneumothorax, or pneumomediastinum in the thoracic follow-up radiographs at the end of the procedure.

The only adverse event recorded was aerophagia in a total of 14/20 dogs. A total of 6/10 dogs in the TOT and 8/10 dogs in the HFOT group showed some degree of aerophagia when comparing radiographs before and after the procedure. In the TOT group, 2/10 dogs had mild and moderate gas distension of the esophagus, 1/10 dogs had mild gastric dilation, and 3/10 dogs showed gas distension of both the esophagus and stomach, with one each having mild, moderate, and severe signs of gas insufflation.

Of the eight dogs in the HFOT group, 4/8 showed esophageal gas distension, 2/8 had gastric dilation, and 2/8 had dilation of both the esophagus and stomach. Among these eight dogs, five showed only mild radiographic signs of aerophagia (3/5 esophagus, 1/5 stomach, 1/5 both locations), while three dogs had severe signs of aerophagia on radiographs, with two experiencing gastric distension and one both gastric and esophageal insufflation (see Supplementary Figure S1).

**Figure 6:**
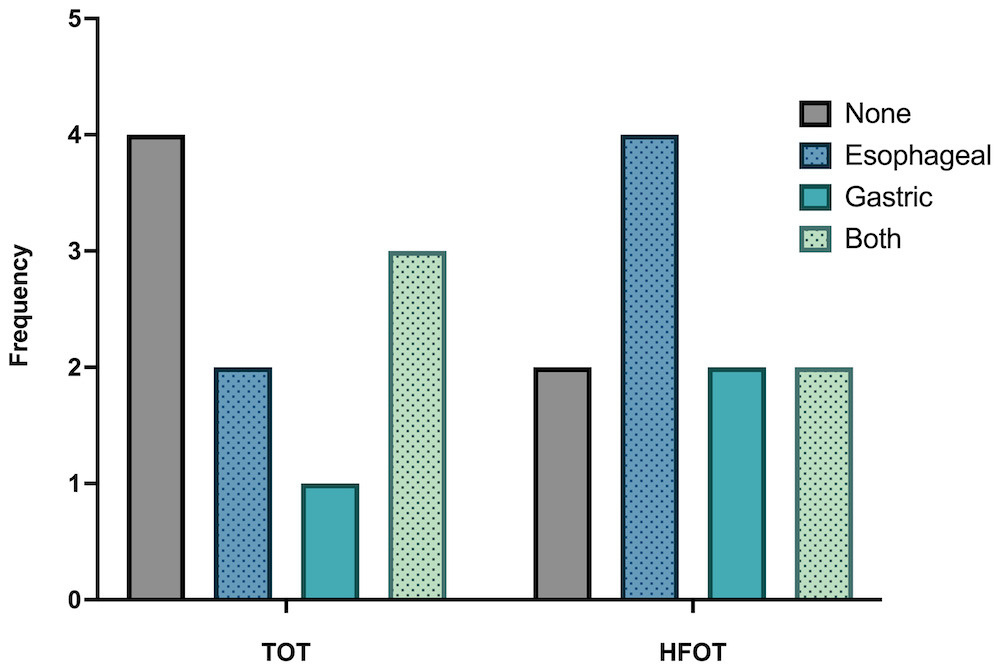
Frequency and distribution of aerophagia compared between the TOT and HFOT groups. TOT, traditional oxygen therapy; HFOT, High-Flow Oxygen Therapy.

Overall, none of the patients experiencing aerophagia had clinical signs at the time of radiographic imaging, nor 1 h after the procedure, and intervention was not deemed necessary in any of the patients. The distribution of aerophagia between both groups is depicted in Figure 6.

## Discussion

The present study demonstrates that HFOT is a feasible and effective method to improve oxygenation in dogs during bronchoscopy. The findings support the hypothesis that HFOT is superior to TOT, with dogs in the HFOT group exhibiting overall higher arterial partial pressure of oxygen and fewer incidents of hypoxemia compared to those in the TOT group. Despite five dogs in the HFOT group being hypoxemic at baseline, only one dog from the group experienced a PaO_2_ below 80 mmHg during the procedure. In contrast, three of the five initially hypoxemic dogs and two dogs with normal baseline values in the TOT group developed hypoxemia with a PaO_2_ below 80 mmHg at least once during bronchoscopy. Although a comparison of both oxygen supplementation methods did not achieve statistical significance at all time points, a consistent trend toward improved oxygenation in the HFOT group could be observed at all times. The study data reflects results from previous studies in people and dogs, making HFOT an alternative for oxygen supplementation during bronchoscopies (14–16, 24, 26–28).

The most notable difference in oxygenation between both methods was visible after preoxygenation. Induction of general anesthesia always carries an increased risk of developing hypoxemia as it leads to respiratory depression, decreased cardiac output, and ventilation-perfusion mismatch caused by hypoventilation or apnea (29, 30). This risk is further exacerbated in patients with pre-existing respiratory diseases affecting pulmonary functions. To prolong the time before desaturation between induction of anesthesia and securing of the airways, preoxygenation with high FiO_2_ concentrations is generally performed (31). Several studies in people have shown that the beneficial effects of HFOT can be used during preoxygenation (32–34) with the main advantage being that oxygen administration can be continued without interruption while the airways are being secured. The findings in the present study reflect previous study results in people, as dogs preoxygenated with HFOT showed significantly higher PaO_2_ after 5 min of preoxygenation than patients in the TOT group. Differences in flow rates and achievable FiO_2_ are the most likely explanations for the improved oxygenation achieved within the HFOT group.

The necessity for general anesthesia and additional diagnostics, particularly BAL sampling, are the main risks for the development of hypoxemia during bronchoscopy in dogs.

Although HFOT managed to prevent hypoxemia in nine out of 10 dogs after induction, with a clear visual and numerical trend toward improved oxygenation, the difference in PaO_2_ between both groups did not reach statistical significance. This could be because two dogs in the TOT group showed an actual increase in PaO_2_ after induction from the measurement obtained after preoxygenation. One possible explanation for this increase is a difference in induction time, allowing for a prolonged preoxygenating effect. Induction was performed slowly and titrated to effect to maintain spontaneous breathing in all patients. Both dogs in the TOT groups might have experienced a more prolonged onset of action with an extended period of less depressive ventilatory effects. As oxygen supplementation continued without interruption, the preoxygenation time would have been prolonged, thus causing the higher PaO_2_ values. However, the duration of induction was not measured to confirm a prolonged preoxygenation period. Both dogs also showed panting during the preoxygenation phase, which may have led to inadequate tidal volume and reduced preoxygenation efficiency, potentially explaining the divergence between measurements at t1 and t2.

To evaluate the effects of BAL on oxygenation, arterial blood gas samples were obtained after visual evaluation of the bronchial tree and immediately before BAL sampling. The bronchoscope was already placed in the designated bronchial segment when the blood sample was drawn; thus, a partial bronchial occlusion occurred in all patients. Although there was no statistically significant difference in PaO_2_ between both groups before BAL sampling, PaO_2_ values were once again higher in the HFOT group.

Three dogs in the TOT group were hypoxemic before BAL; two were the smallest patients in the group, and one was even the smallest dog in the entire patient population. Thus, body size was likely associated with these findings, with a higher degree of airway obstruction by the bronchoscope occurring in smaller patients, hindering sufficient oxygen administration. As hypoxemia did not occur in the smallest dog in the HFOT group, the differences in flow rate and FiO_2_ between both methods might have played a key role. Lower flow rates applied with TOT might not have been sufficient to create high enough FiO_2_ to overcome the desaturation caused by the airway obstruction with the bronchoscope. However, the smallest dog in both the TOT group and the entire patient population was already hypoxemic at baseline and also failed to show improvement after preoxygenation and after removing the bronchoscope. This failure to adequately respond to oxygen administration might have also resulted from an underlying pathology, as the dog showed an elevated AaDO_2_ difference at baseline.

Additionally, this dog showed excessive mucus production, which might have simply acted as a mechanical barrier, further obstructing the already narrowed tracheal lumen due to the presence of the bronchoscope. Therefore, alveolar collapse and a V/Q mismatch due to obstruction with mucus or secretions might be another possible explanation for the failure to improve despite oxygen therapy.

In the HFOT group, the dog diagnosed with pulmonary fibrosis and bronchial collapse was the only patient with a PaO_2_ below 80 mmHg before BAL sampling. Respiratory compromise caused by the underlying pulmonary fibrosis might have been further pronounced due to bronchial collapse, which in turn might have been exacerbated due to local irritation from the bronchoscope. None of the remaining dogs in the HFOT group experienced PaO_2_ below 133 mmHg, with two dogs achieving values above 300 and four above 400 mmHg.

BAL sampling has been shown to cause worsening of hypoxemia during bronchoscopy in people (35, 36), and a decrease in PaO_2_ after BAL was expected to occur in the present study population. The main question was if HFOT could prevent critically low levels of PaO_2_ during this phase of bronchoscopy. While two dogs in the TOT group experienced hypoxemia below 80 mmHg, only one dog in the HFOT group experienced a decrease in PaO_2_ below 80 mmHg after BAL. This patient was once again the dog suffering from pulmonary fibrosis. After BAL sampling, this particular dog experienced severe hypoxemia with a PaO_2_ below 50 mmHg. In retrospect, this value would have called for intervention and escalation to advanced oxygen therapy in the form of assisted ventilation. However, continuous monitoring showed only a brief few-second drop of SpO_2_ to a value below 90%, and the dog had no visible cyanosis or other clinical signs that would have prompted immediate intervention or escalation of therapy, at the time. Due to the delay caused by the duration of the blood gas measurement, these low values were not immediately recognized at the time of BAL sampling. By the time sample analysis was completed, the bronchoscope had already been removed from the airways. The choice to no longer intervene was further confirmed by the following blood gas sample, showing an elevation of the PaO_2_ above 400 mmHg.

While HFOT improved oxygenation in nine out of 10 patients after BAL sampling, the results obtained in this one dog question if HFOT is also suitable for patients with severe hypoxemia or if other oxygenation methods should be employed in these patients. Similar concerns were raised by the authors of two studies in people evaluating HFOT compared to NIV during bronchoscopy (27, 37). Both studies concluded that HFOT should not be used in people with severe hypoxemia as it cannot provide controlled ventilatory support, and while increases in airway pressure are reported, they remain unpredictable and non-adjustable. It is possible that simply increasing the flow rate would have been sufficient to improve oxygenation in this particular dog, as hypoxemia most likely resulted from an altered gas exchange due to pulmonary fibrosis and airway collapse. Higher flow rates might have been able to create a PEEP effect, hindering the collapse of smaller airways and increasing FiO_2_. Based on the results of this dog, care should be taken in patients with severe hypoxemia present at baseline, and alternative oxygen supplementation methods should be considered in these patients.

Despite this one severely hypoxemic patient, it is important to note that none of the other dogs in the HFOT group developed hypoxemia. The lowest PaO_2_ recorded in this group was 140 mmHg, with a mean PaO_2_ of 305 mmHg, compared to a mean of 192 mmHg in the TOT group, where three dogs showed values below 100 mmHg. A possible explanation for this difference in observation can be the combination of higher FiO_2_ and higher flow rates, creating enough airway pressure to prevent alveolar collapse during BAL sampling.

As this is the first known study to evaluate serial arterial blood gas analyses during bronchoscopy in dogs, what can be gathered from the findings is that despite all preparations and initial improvements of oxygenation, bronchoscopy and, in particular, bronchoalveolar lavage can cause severe deterioration of oxygenation, especially in already compromised patients. Ideally, a patient's pulmonary function should be evaluated before the procedure, as all patients who deteriorated during bronchoscopy showed hypoxemia at baseline, regardless of their group allocation.

Arterial blood gas measurements demonstrated the beneficial effects of HFOT on oxygenation during bronchoscopy. They also confirmed that alterations in pulmonary function and occurrences of hypoxemia caused by BAL sampling are temporary and that the effects of oxygen supplementation are short-lived, diminishing once oxygen administration is discontinued. However, nearly all patients showed slightly higher PaO_2_ values and lower AaDO_2_ ratios 1 h after bronchoscopy compared to values obtained at baseline (see Supplementary Figure S2).

Although the reason for this observation is unknown, it is possible that either sufficient positive airway pressure to cause alveolar recruitment during the procedure occurred or that suctioning of BAL fluid also removed build-up mucus in lower airways, resulting in clearance of the airways with subsequent improved ventilation/perfusion. Brach et al. (38) also observed an improvement in regional V/Q immediately after bronchoscopy in people using scintiphotography. Removal of mucus plugs and thick secretions was noted in all affected patients and was likely the reason for the observed improvement.

Severe complications associated with HFOT, such as pneumothorax or pneumomediastinum, are rare, and the results of the present study reflect previous findings, as none of the dogs had clinical or radiographic signs of pneumothorax or pneumomediastinum at the end of the procedure.

The present study's main adverse event was the occurrence of aerophagia in a total of 14 dogs. Aerophagia was also the most common adverse event reported in three other veterinary studies (11, 18, 19). In the study by Jagodich et al. (11), the entire patient population of eight dogs experienced aerophagia after receiving HFOT. However, radiographic images were not performed after each HFOT flow rate change or after receiving conventional oxygen therapy.

In the present study, six dogs in the TOT group and eight in the HFOT group showed some degree of aerophagia at the end of the bronchoscopic procedure. Although most cases showed only mild signs, three dogs in the HFOT group and one in the TOT group had severe signs of gaseous distension. The precise cause of aerophagia in these patients is unknown but might be explained by several factors. It is possible that in the present study population, HFOT created high enough pharyngeal airway pressures to dilate the esophagus, inadvertently insufflating the gastrointestinal tract. A case report on gastric distension in a pediatric patient after using HFOT (39) argued that too deeply inserted nasal cannulas likely caused high enough pharyngeal airway pressure to distend the esophageal sphincter, resulting in gastric distension. In the present study population, care was taken to choose the correct nasal cannula size, occluding no more than 50% of the nares to avoid excessive airway pressures. However, it cannot be ruled out for certain that some cannulas still occupied too much internal, non-visible space of the nares. Different studies showed a linear relationship between flow rate and pharyngeal pressures in patients receiving HFOT (40–42). Therefore, larger dogs with correspondingly higher flow rates might have experienced higher pharyngeal airway pressures opening the esophageal sphincter. However, most studies reported high pressures only for closed mouth conditions in people (40, 41, 43, 44), and as all dogs in the present study were positioned with their mouths ajar, it is unclear whether sufficient pharyngeal pressures were generated to have made splinting and insufflation of the esophagus possible. Unfortunately, pharyngeal airway pressure was not measured in the present study. Future studies evaluating the correlation of airway pressures with occurrences and severity of aerophagia in dogs might thus be beneficial in gaining a better understanding of the reasons behind aerophagia in dogs receiving High-Flow Oxygen Therapy.

Additionally, physiological alterations due to general anesthesia might have also been responsible for aerophagia in some patients. On the one hand, as spontaneous breathing had to be maintained throughout the procedure, some patients might have shown increased swallowing of air due to insufficient anesthetic depth, although this was not clinically detected in any patient. On the other hand, possible anesthesia-related reduced esophageal sphincter tone might have also played a role (45–47). Low doses of propofol have been shown to cause a reduction in upper esophagus sphincter tone in people (46), while a large group of anesthetic drugs can affect the lower esophageal sphincter (45, 47). However, since aerophagia also occurred in studies using HFOT in awake dogs, it is more likely that aerophagia is related to the high flow rates *per se* and the resulting airway pressures. Therefore, aerophagia might have already occurred during the phase of preoxygenation, when patients either had their mouths closed, thus possibly achieving higher airway pressures, or showed panting, which might have caused increased swallowing of air.

Although radiographs showed severe gastric and/or esophageal distension in four dogs, none of the patients showed clinical signs such as abdominal pain, regurgitation, or discomfort, thus, an intervention was not deemed necessary in any patient. In addition, none of the dogs showed clinical signs or the need for intervention when reassessed during blood sampling 1 h after the procedure.

As aerophagia also occurred in patients receiving TOT, monitoring is warranted for all patients receiving oxygen supplementation and further studies in dogs evaluating the occurrence of aerophagia in relation to oxygen therapy in general are needed to better understand its mechanisms.

The present study has some limitations. First, the patient population is heterogeneous in terms of underlying diseases. The diversity of underlying respiratory conditions among the dogs included in the study could have influenced their response to oxygen therapy, making it difficult to draw conclusions about the efficacy of HFOT in specific disease states. A more homogenous study population would have been beneficial in confirming these findings and exploring the role of HFOT in dogs with specific respiratory pathologies. However, since bronchoscopy was part of the diagnostic workup, final diagnoses could not be established before study inclusion. Therefore, the arterial partial pressure of oxygen was measured, and AaDO_2_ was calculated for each patient at baseline, which allowed evaluation and comparison of pulmonary function between the patients. Both groups had no significant difference between baseline PaO_2_, AaDO_2_, or any other physiological parameter, creating an ideal basis for comparing responses to the different oxygen supplementation methods. Nevertheless, differences in severity and pathomechanisms of the underlying conditions might have affected oxygenation and response to oxygen therapy.

Another limitation of the study is the small sample size. The initial target was 25 dogs per group. This target was deemed achievable based on a retrospective analysis of patient numbers (~80–100 dogs/year) presented for bronchoscopy in the previous 3 years before data collection commenced. Unfortunately, from May 2020 until August 2022, only 23 patients eligible for study inclusion were presented, of which three dogs had to be excluded. One possible explanation for the sudden drop in patient numbers could be the corona pandemic resulting in lockdown periods, travel restrictions, financial challenges, and changes in our hospital admission procedures. However, the actual reasons for the decline in patient numbers remain unknown and are most likely due to a combination of multiple factors. The small sample size might have also affected the outcome of occurring complications. Air leak syndrome is already a rare occurrence in people (48), so the study might have simply been underpowered to detect such rare complications associated with HFOT.

Flow settings in the HFOT group were based on recommendations of a previous study evaluating HFOT in healthy dogs (11). However, one dog in the HFOT group of the present study received a lower flow rate than previously planned due to its body weight and flow rate limitations of the Vapotherm Precision Flow Device. Although this dog showed initial hypoxemia, PaO_2_ values increased after administration of HFOT, so this patient was not excluded from the study despite receiving a lower flow rate, as this exception was deemed unlikely to affect overall results. Furthermore, since the present study only evaluated one flow rate setting for the HFOT group, it remains unknown if higher flow rates might have prevented hypoxemia even in the severely hypoxemic dog and if it would have led to statistically significant differences compared to TOT at all time points. Future studies evaluating different flow rates in larger patient populations might prove helpful in answering still-open questions.

In conclusion, the present study shows that HFOT is a safe and effective method to improve oxygenation during bronchoscopy in dogs. Although TOT via nasal cannulas managed to provide adequate oxygen supplementation in some patients, HFOT proved to be statistically superior for preoxygenation (t1) and at the end of the procedure (t5). While statistical significance was not achieved at all time points, including after BAL where it was narrowly missed, visual analysis of the data indicates a clear trend toward improved oxygenation with HFOT throughout the study. Overall, the data suggests fewer incidents of hypoxemia during bronchoscopy and BAL sampling in the HFOT group. While HFOT showed clear advantages in oxygenation compared to TOT in patients experiencing mild to moderate hypoxemia at baseline, caution is advised in severely hypoxemic patients. Alternative oxygenation strategies such as non-invasive ventilation (NIV) or assisted ventilation should be considered in these cases, as HFOT remains an open system relying on the patient's spontaneous breathing.

## Data Availability

The raw data supporting the conclusions of this article will be made available by the authors, without undue reservation.
