# Supplementary figures and images for: Effects of high-flow oxygen therapy on oxygenation in dogs undergoing diagnostic bronchoscopy
Source: Front Vet Sci. 2025 Mar 24;12:1545427. doi: 10.3389/fvets.2025.1545427 (PMC11974253; doi:10.3389/fvets.2025.1545427)

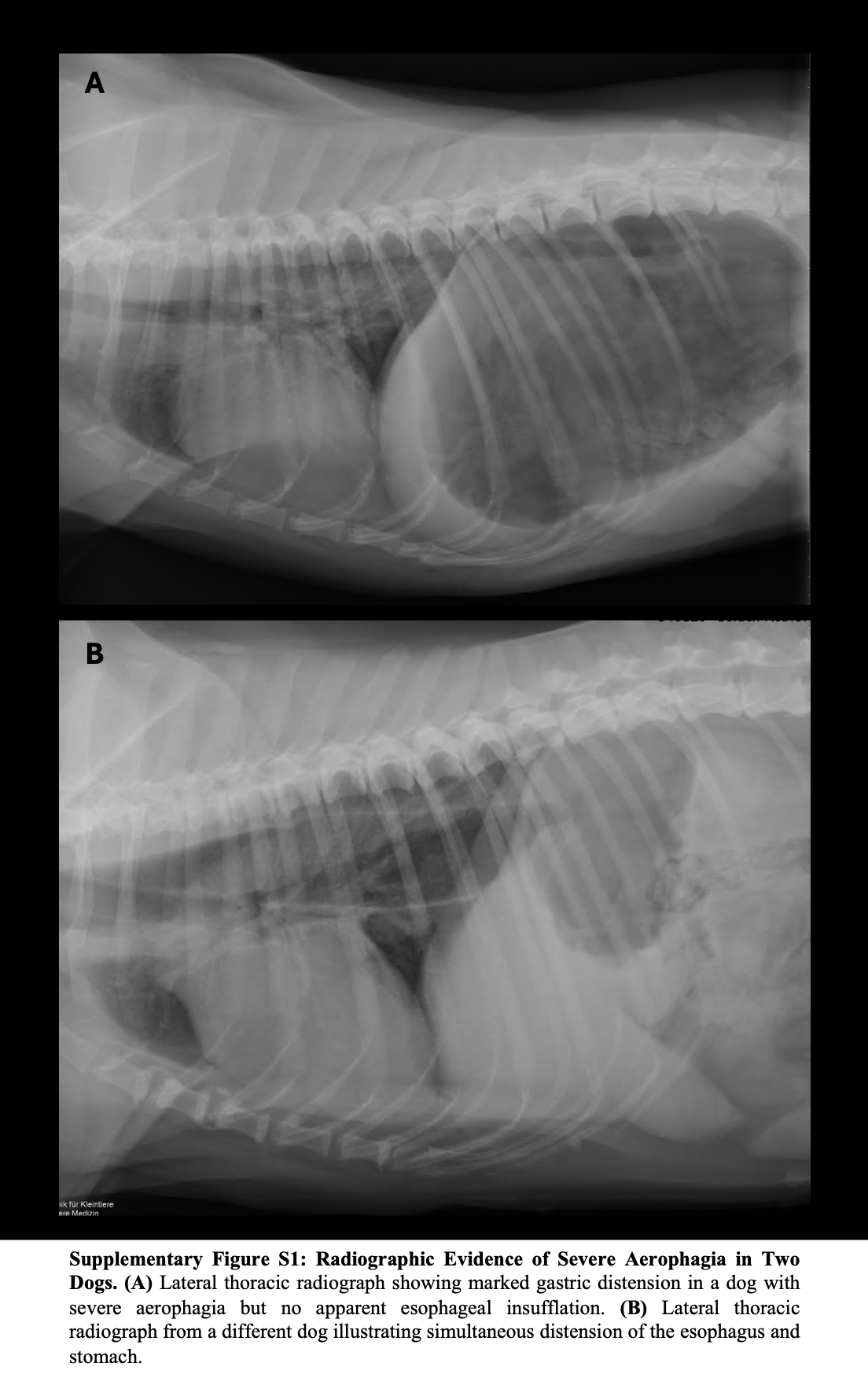

Supplement: Supplementary file 1 [file Image_1.tiff]

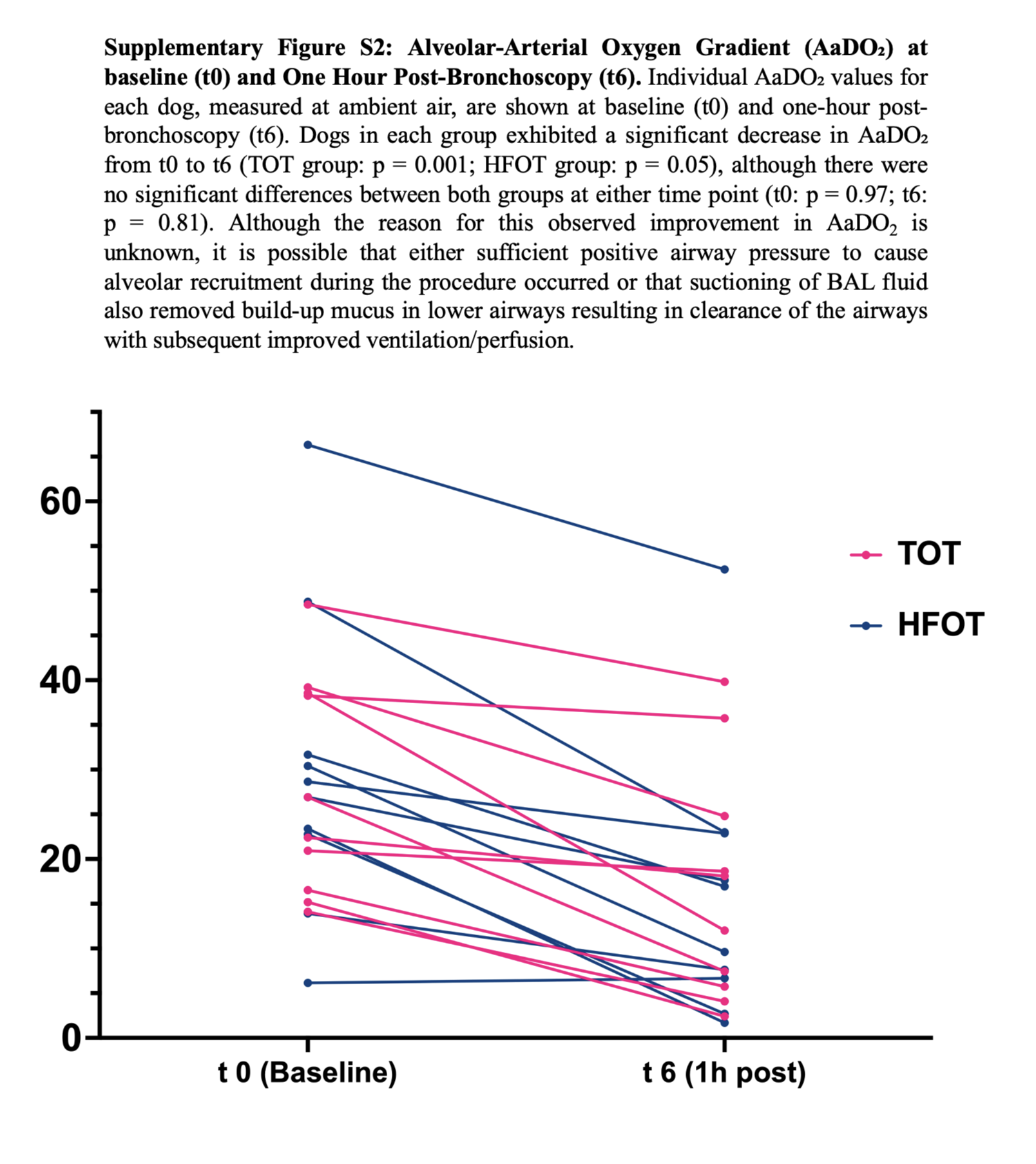

Supplement: Supplementary file 2 [file Image_2.tiff]
